# Diffusion‐weighted MRI and intravoxel incoherent motion model for diagnosis of pediatric solid abdominal tumors

**DOI:** 10.1002/jmri.25901

**Published:** 2017-11-21

**Authors:** Emma M. Meeus, Niloufar Zarinabad, Karen A. Manias, Jan Novak, Heather E.L. Rose, Hamid Dehghani, Katharine Foster, Bruce Morland, Andrew C. Peet

**Affiliations:** ^1^ Physical Sciences of Imaging in Biomedical Sciences (PSIBS) Doctoral Training Centre University of Birmingham UK; ^2^ Institute of Cancer and Genomic Sciences University of Birmingham UK; ^3^ Department of Oncology Birmingham Children's Hospital Birmingham UK; ^4^ School of Computer Science University of Birmingham UK; ^5^ Department of Radiology Birmingham Children's Hospital Birmingham UK

**Keywords:** abdominal tumors, diffusion, diffusion‐weighted imaging, IVIM, pediatric, perfusion

## Abstract

**Background:**

Pediatric retroperitoneal tumors in the renal bed are often large and heterogeneous, and their diagnosis based on conventional imaging alone is not possible. More advanced imaging methods, such as diffusion‐weighted (DW) MRI and the use of intravoxel incoherent motion (IVIM), have the potential to provide additional biomarkers that could facilitate their noninvasive diagnosis.

**Purpose:**

To assess the use of an IVIM model for diagnosis of childhood malignant abdominal tumors and discrimination of benign from malignant lesions.

**Study Type:**

Retrospective.

**Population:**

Forty‐two pediatric patients with abdominal lesions (*n* = 32 malignant, *n* = 10 benign), verified by histopathology.

**Field Strength/Sequence:**

1.5T MRI system and a DW‐MRI sequence with six *b*‐values (0, 50, 100, 150, 600, 1000 s/mm^2^).

**Assessment:**

Parameter maps of apparent diffusion coefficient (ADC), and IVIM maps of slow diffusion coefficient (*D*), fast diffusion coefficient (D*), and perfusion fraction (*f*) were computed using a segmented fitting model. Histograms were constructed for whole‐tumor regions of each parameter.

**Statistical Tests:**

Comparison of histogram parameters of and their diagnostic performance was determined using Kruskal–Wallis, Mann–Whitney *U*, and receiver‐operating characteristic (ROC) analysis.

**Results:**

IVIM parameters D* and *f* were significantly higher in neuroblastoma compared to Wilms' tumors (*P* < 0.05). The ROC analysis showed that the best diagnostic performance was achieved with D* 90^th^ percentile (area under the curve [AUC] = 0.935; *P* = 0.002; cutoff value = 32,376 × 10^−6^ mm^2^/s) and *f* mean values (AUC = 1.00; *P* < 0.001; cutoff value = 14.7) in discriminating between neuroblastoma (*n* = 11) and Wilms' tumors (*n* = 8). Discrimination between tumor types was not possible with IVIM *D* or ADC parameters. Malignant tumors revealed significantly lower ADC, *D*, and higher D* values than in benign lesions (all *P* < 0.05).

**Data Conclusion:**

IVIM perfusion parameters could distinguish between malignant childhood tumor types, providing potential imaging biomarkers for their diagnosis.

**Level of Evidence:** 4

**Technical Efficacy:** Stage 2

J. Magn. Reson. Imaging 2018;47:1475–1486.

Malignant abdominal tumors in children are often diagnosed using a combination of conventional imaging and histology.^1^, ^2^ Histological diagnosis requires an invasive biopsy, with risk of morbidity and sampling error in large heterogeneous lesions such as seen in the abdomen.^3^, ^4^ A conclusive diagnosis based on conventional imaging alone can be difficult, with similar morphological appearances of some childhood tumors.^5^

Magnetic resonance imaging (MRI) is increasingly used in the diagnosis, staging, and management of pediatric solid tumors due to its relatively high resolution and lack of nonionizing radiation.^6^ Diffusion‐weighted MRI (DW‐MRI) has shown promising results with the use of apparent diffusion coefficient (ADC) maps. Previous studies have demonstrated that ADC can discriminate benign from malignant solid tumors,^7^, ^8^ which is likely due to the proposed inverse relationship between ADC and cellularity.^9^, ^10^ ADC has been determined to be lower in pediatric malignant abdominal tumors in comparison to benign lesions, relating to the more restricted diffusion and hence higher cellularity.^7^ Noninvasive discrimination between individual tumor types has not been possible in previous pediatric studies, but would be of considerable clinical value.

The ADC approach for heterogeneous tissues such as those found in abdominal tumors can be relatively simplistic and does not maximize the information that can be potentially extracted from the DWI.^11^ The intravoxel incoherent motion (IVIM) model with multi *b*‐value DW‐MRI can account for the pure diffusion characteristic (*D*), and separate the pseudodiffusion (D*) effect caused by microcirculation or blood perfusion, and determine the perfusion fraction (*f*) corresponding to the fraction of signal arising from the vascular component.^12^, ^13^ Both diffusion characteristics influence the measured diffusion‐weighted signal and therefore limit the reliability of the ADC measurement. Previous studies have shown IVIM parameters to be helpful in discriminating common adult malignant pancreatic tumors as well as benign from malignant lesions.^14^, ^15^ Significantly better discrimination of low‐ and high‐grade hepatocellular carcinoma (HCC) has been demonstrated with IVIM‐*D* in comparison to ADC.^16^

The heterogeneous nature of abdominal tumors can be difficult to characterize based on imaging measures such as the mean ADC value alone.^17^ Histograms allow the inspection of the distribution of values corresponding to a region‐of‐interest (ROI), and describe the statistical information contained within the imaged region. However, the placing of a single ROI on a representative tumor image can lead to sampling bias and not provide accurate representation of the tumor heterogeneity.^18^, ^19^ Alternatively, a whole‐tumor ROI approach has been shown to largely reduce the sampling bias and to produce excellent interobserver agreement compared to single‐slice ROI analysis.^20^ Such an approach was also used in a study of Wilms' tumors, which was able to identify distinct cellular regions based on ADC histograms, and to determine the predominant histological cell types.^21^ Further value of histogram analysis was shown in a study with adult patients with glioblastoma, where the IVIM parameter histograms were able to differentiate between recurrent tumor and treatment effects.^22^

Therefore, the aim of this study was to retrospectively evaluate the feasibility and diagnostic potential of using IVIM and ADC histogram analysis for discriminating between individual malignant and between benign and malignant pediatric tumor types.

## Materials and Methods

### Study Population

**Figure 1:**
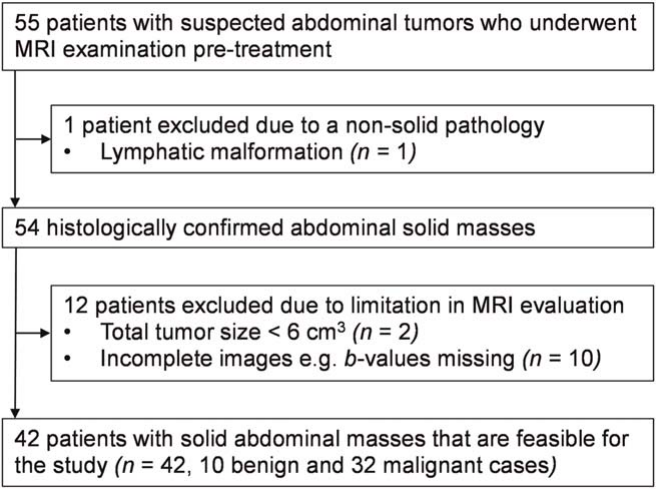
Flow diagram for patient selection based on the recommended STARD standards for reporting diagnostic accuracy.

The protocol for this retrospective study was approved by the East Midlands–Derby Research Ethics Committee (REC 04/MRE04/41), operating under the rules of Declaration of Helsinki 1975 (and as revised in 1983). Informed parental consent was obtained from all subjects after the image acquisition. A computerized search of medical records identified 55 patients who underwent abdominal MRI including DWI between June 2012 and September 2016. All cases were reviewed by the tumor study board for suspected solid malignancy. The following exclusion criteria were applied to the cohort: having received treatment prior to imaging (chemotherapy or surgery), nonsolid pathology (*n* = 1), incomplete or improper MRI scans (*n* = 10), or small lesions where single‐slice largest area <3 cm^2^ or total volume <6 cm^3^ (*n* = 2). The final population included 42 patients, comprised of 10 benign and 32 malignant cases (Fig. 1).

The cohort comprised 16 female patients (age range, 0–14 years; mean age ± standard deviation [SD], 3.6 ± 4.2) and 26 male patients (age range, 0–10 years; mean age ± SD, 3.3 ± 3.0). The age range for the whole group was 0–14 years, with a mean age ± SD 3.7 ± 2.8 years. The malignant cases included those with clear cell sarcoma (*n* = 1), Ewing's sarcoma (*n* = 1), germ cell tumor (*n* = 1), hepatoblastoma (*n* = 4), nephroblastomatosis (*n* = 1), neuroblastoma (*n* = 11), ovarian immature teratoma (*n* = 1), rhabdoid (*n* = 2), rhabdomyosarcoma (*n* = 2), and Wilms' tumor (*n* = 8). The benign lesions consisted of indolent abdominal mass (*n* = 1), ganglioneuroma (*n* = 3), hematocolpos (*n* = 1), lipoma (*n* = 1), hemangioma (*n* = 1), mesoblastic nephroma (*n* = 1), osteomyelitis (*n* = 1), and vascular malformation (*n* = 1). The mean size of the malignant tumors was 32.1 cm (range 8.5 ± 53.1 cm) and benign lesions 16.8 cm (range 7.2 ± 47.7 cm). The cohort demographics are summarized in Table 1.

**Table 1:** Patient Cohort Demographics

Lesion	Cases	Mean age, range (yrs)	Sex (F/M)
Benign	10	4 (0‐10)	3/7
Clear cell sarcoma	1	3.5	0/1
Ewing's sarcoma	1	9.3	0/1
Germ cell	1	2.4	0/1
Hepatoblastoma	4	0.9 (0‐2)	2/2
Nephroblastomatosis	1	0.1	0/1
Neuroblastoma	11	1.9 (0‐6)	5/6
Ovarian immature teratoma	1	11.8	1/0
Rhabdoid	2	0.9 (0‐1)	1/1
Rhabdomyosarcoma	2	5.8 (5‐7)	0/2
Wilms' tumor	8	6.1 (1‐14)	4/4
Total	42	3.7 (0‐14)	16/26

### MRI

MRI was performed with a Siemens Avanto 1.5T (Siemens Healthcare, Erlangen, Germany) scanner and a 4‐channel body receive coil at Birmingham Children's Hospital. The imaging protocol included fat‐suppressed axial and coronal pre‐ and postgadolinium T_1_‐weighted turbo spin‐echo (repetition time / echo time [TR/TE] 760 to 817/7.7 msec), axial T_2_‐weighted turbo spin‐echo (TR/TE 3000 to 5640/67 to 87 msec) and DWI acquisition. The DWI protocol used a spin‐echo planar imaging (EPI) sequence with six *b*‐values (0, 50, 100, 150, 600, 1000 s/mm^2^), EPI factor = 174, three averages (NSA = 3), TR/TE 3200 to 9900/92 msec, parallel imaging (GRAPPA) with an acceleration factor of two, and 75% partial Fourier encoding. The diffusion‐weighting was applied in three orthogonal directions, of which an average image was derived in the axial acquisition plane. Depending on patient size, the field‐of‐view (FOV) was 221 to 350 × 172 to 317 mm, matrix size 122 to 192 × 128 to 192, slice thickness 5 mm with no gap between slices and voxel size of 1 to 2.34 mm^2^ × 5 mm. The DWI protocol acquisition time ranged from 4 min 52 sec to 7 min 35 sec.

### IVIM Modeling of the DW Data and Computation of ADC

Postprocessing of the DW MR data was performed using an in‐house imaging analysis tool developed in MeVisLab platform (v. 2.7.1, MeVis Medical Solutions, Bremen, Germany). The analysis algorithm for IVIM was developed in Python (v. 2.7). The relationship between the diffusion signal intensities and the *b*‐values can be described by a biexponential relationship introduced by Le Bihan^12^:

(1) $$S_{b}/S_{0}=f\cdot \text{exp}\left(-bD*\right)+\left(1-f\right)\cdot \text{exp}\left(-bD\right)$$

where *S*_0_ and *S*_b_ are the signal intensities at *b* = 0 and *b* = 50, 100, 150, 600, or 1000 s/mm^2^, respectively. Using this relationship, a nonlinear least‐squares fit was applied to the data on a voxel‐by‐voxel basis. A stepwise fitting was used to increase the stability of the biexponential fitting and the reliability of the IVIM parameters as reported by previous studies.^23^, ^24^, ^25^, ^26^ At high *b*‐values >100 s/mm^2^ the perfusion effects are assumed negligible as D* ≫*D*, and linear regression can be performed to compute the *D* parameter from the gradient of the fit.^27^ The same fit can be used to deduce the *f* parameter from extrapolating the fit to the *y*‐axis and taking the difference to the *b*_0_ signal. The biexponential fitting was then performed using the Levenberg–Marquardt algorithm with the predefined values of *D* and *f* to find the D* parameter.

The computation of ADC values was performed using a monoexponential linear fit of the *b*‐values 0 and 1000 s/mm^2^:

(2) $$\frac{S_{b}}{S_{0}}=\text{exp}\left(-b\cdot ADC\right)$$

The ADC and IVIM parameter maps were analyzed using in‐built histogram and ROI drawing modules. For each case, mean, median, 5^th^, 25^th^, 75^th^, and 90^th^ percentiles, skewness, kurtosis, and entropy were calculated from the normalized histograms based on values extracted from the ROI. The histograms were normalized to the maximum value due to differences in ROI sizes, and to compute average histograms for individual tumor types.

Whole tumor areas were included in the ROIs, including cystic and necrotic areas. The ROIs were drawn manually on DW *b* = 0 images by an author with clinical experience (K.M.), after which they were refined by a consultant radiologist (K.F., 10 years experience in abdominal MRI). Any changes to the ROIs were made in consensus of the two authors (K.M. and K.F.). Conventional MR images (T_1_ and T_2_) were used to aid delineation of tumors at each consecutive slice, excluding peritumoral edema. On each slice, sections of tumor that were >50% of the more central adjacent slice and ≥3 cm were included to minimize any partial volume effects.

### Statistical Analysis

All statistical analyses were performed using SPSS Statistics (v. 23, Chicago, IL) software. A nonparametric Kruskal–Wallis test was used for the comparison of IVIM and ADC histogram parameters from the malignant tumors and Dunn's test was performed to determine which tumors gave rise to the difference. Mann–Whitney *U*‐test was used to establish differences between the malignant and benign lesions. Bonferroni correction was used.

Receiver operating characteristic (ROC) curves were used to determine how well the diffusion parameters discriminated individual tumor types, and benign from malignant lesions. The cutoff value that demonstrated the greatest Youden index on the estimated curves was used to determine the sensitivity and specificity. The area under the ROC curve (AUC) was determined to assess the diagnostic performance of the IVIM and ADC histogram parameters.

Voxelwise correlations between perfusion fraction and diffusion parameters were assessed for whole tumor ROIs and tumor regions where *f* was between 25–40%, with Pearson correlation coefficient, *r*. This was performed for cases of neuroblastoma (*n* = 5) and Wilms' tumors (*n* = 4).

All tests performed were two‐sided. Numerical values are reported as median ± SD. *P* < 0.05 was considered to indicate a significant difference.

## Results

### Discrimination Between Tumor Types

**Figure 2:**
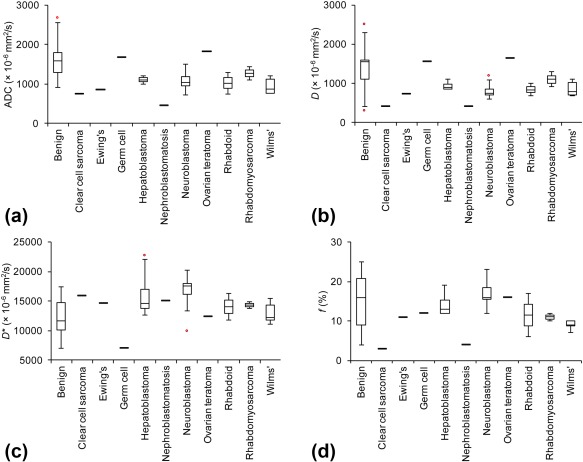
Boxplot distributions of median ADC and IVIM parameters for different tumor types. Plots shown for **(a)** ADC, **(b)** *D*, **(c)** D*, and **(d)** *f* parameter. Top and bottom of boxes represent 25% and 75% percentiles of data values, respectively, and the horizontal lines in boxes represent the median. The whiskers extend to the most extreme data points not considered outliers and outliers are indicated by red circles.

Distributions of median ADC and IVIM parameter values of malignant and benign lesions are presented in Fig. 2 for our whole patient cohort.

Comparison between the individual malignant tumor types and their ADC, D, D*, and *f* histogram parameters are presented in Table 2 with the histogram parameter values summarized in Table 3. The Kruskal–Wallis test revealed significant differences in the histogram parameters of D* (*P* 0.007–0.021) and *f* (*P* < 0.001–0.008) between the malignant tumors. For both D* and *f* these included histogram parameters mean, median, and 75^th^/90^th^ percentiles. Additionally, D* skewness and *f* 25^th^ percentile, kurtosis, and entropy showed significant differences. No differences were observed for ADC and *D* histogram parameters. The Dunn's test revealed that the D* values of neuroblastoma were significantly higher in comparison to Wilms' tumor (*P* 0.005–0.012) and skewness was significantly higher for Wilms' (*P* = 0.017) compared to neuroblastoma. Similarly, the *f* values in neuroblastoma were significantly higher in comparison to Wilms' tumors (*P* < 0.001–0.002) and higher kurtosis (*P* = 0.006) and entropy (*P* < 0.001) were seen for neuroblastoma.

**Table 2:** Comparison of Malignant Tumor Types

Parameter	Kruskal‐Wallis test (*P* value)				Dunn's test (*P* value)			
	ADC	*D*	D*	*f*	ADC	*D*	D*	*f*
Mean	0.470	0.277	0.013	< 0.001	—	—	NB‐W = 0.010	NB‐W < 0.001
Median	0.273	0.198	0.013	< 0.001	—	—	NB‐W = 0.010	NB‐W < 0.001
5^th^ percentile	0.201	0.088	0.944	0.110	—	—	—	—
25^th^ percentile	0.151	0.142	0.413	0.003	—	—	—	NB‐W = 0.002
75^th^ percentile	0.244	0.340	0.015	< 0.001	—	—	NB‐W = 0.012	NB‐W < 0.001
90^th^ percentile	0.392	0.405	0.007	0.001	—	—	NB‐W = 0.005	NB‐W < 0.001
Kurtosis	0.395	0.445	0.651	0.008	—	—	—	NB‐W = 0.006
Skewness	0.183	0.344	0.021	0.651	—	—	NB‐W = 0.017	—
Entropy	0.462	0.466	0.974	0.001	—	—	—	NB‐W < 0.001

HB: Hepatoblastoma; NB: Neuroblastoma; W: Wilms' tumor; with ADC, D, D*, and *f* histogram parameters.

**Table 3:** Histogram Parameters of ADC, D, D*, and *f* for the Individual Malignant Tumor Types

		Mean	Median	5^th^ %	25^th^ %	75^th^ %	90^th^ %	Kurtosis	Skewness	Entropy
HB *n* = 4	ADC	1181 ± 71	1104 ± 77	767 ± 110	972 ± 66	1301 ± 96	1632 ± 96	2.19 ± 4.0 × 10^−2^	3.1 × 10^−2^ ± 1.6 × 10^−3^	6.91 ± 2.2 × 10^−1^
	*D*	962 ± 61	938 ± 51	386 ± 131	791 ± 60	1096 ± 65	1356 ± 71	2.21 ± 6.5 × 10^−3^	3.4 × 10^−2^ ± 3.4 × 10^−4^	7.01 ± 8.4 × 10^−2^
	D*	18723 ± 1878	16144 ± 1933	5068 ± 589	11042 ± 1295	24000 ± 2616	33940 ± 3412	2.22 ± 3.0 × 10^−2^	7.3 × 10^−3^ ± 4.3 × 10^−4^	9.78 ± 1.8 × 10^−1^
	*f*	16.6 ± 1.53	14.3 ± 1.43	4.00 ± 0.94	9.00 ± 1.17	20.8 ± 1.78	31.3 ± 2.48	2.15 ± 2.8 × 10^−2^	2.2 × 10^−1^ ± 8.4 × 10^−3^	3.60 ± 7.0 × 10^−2^
NB *n* = 11	ADC	1164 ± 190	1070 ± 201	625 ± 124	825 ± 139	1416 ± 271	1770 ± 352	2.22 ± 7.5 × 10^−2^	3.3 × 10^−2^ ± 5.7 × 10^−3^	7.09 ± 3.9 × 10^−1^
	*D*	885 ± 88	817 ± 85	374 ± 102	616 ± 76	1098 ± 114	1413 ± 157	2.15 ± 5.7 × 10^−2^	3.0 × 10^−2^ ± 6.4 × 10^−3^	7.04 ± 2.3 × 10^−1^
	D*	19348 ± 1213	16957 ± 1318	5120 ± 608	10651 ± 1457	24127 ± 1762	35328 ± 2118	2.22 ± 1.6 × 10^−2^	7.3 × 10^−3^ ± 1.9 × 10^−4^	9.30 ± 5.6 × 10^−1^
	*f*	21.1 ± 1.85	17.1 ± 1.75	3.82 ± 0.47	9.72 ± 1.09	28.6 ± 3.04	43.1 ± 4.27	2.20 ± 1.9 × 10^−2^	2.1 × 10^−1^ ± 6.4 × 10^−3^	3.88 ± 1.2 × 10^−1^
W *n* = 8	ADC	1033 ± 191	939 ± 178	643 ± 103	795 ± 138	1180 ± 243	1511 ± 317	2.19 ± 5.4 × 10^−2^	3.5 × 10^−2^ ± 2.9 × 10^−3^	6.95 ± 3.2 × 10^−1^
	*D*	935 ± 94	847 ± 85	586 ± 42	722 ± 63	1060 ± 120	1368 ± 167	2.19 ± 3.1 × 10^−2^	3.4 × 10^−2^ ± 1.7 × 10^−3^	6.86 ± 2.0 × 10^−1^
	D*	14944 ± 791	13004 ± 787	4762 ± 729	9429 ± 655	18147 ± 1104	25530 ± 1683	2.21 ± 1.2 × 10^−2^	7.8 × 10^−3^ ± 3.8 × 10^−5^	9.84 ± 7.5 × 10^−2^
	*f*	11.0 ± 0.80	9.00 ± 0.50	2.75 ± 0.41	6.50 ± 0.50	13.1 ± 0.92	20.3 ± 2.69	2.07 ± 3.6 × 10^−2^	1.9 × 10^−1^ ± 2.9 × 10^−2^	3.60 ± 7.0 × 10^−2^

HB: Hepatoblastoma; NB: Neuroblastoma; W: Wilms' tumor. Data presented correspond to mean values ± standard deviations. The units for mean, median, and percentiles of ADC, *D*, and D* are in × 10^−6^ mm^2^/s and *f* as a percentage (%).

**Figure 3:**
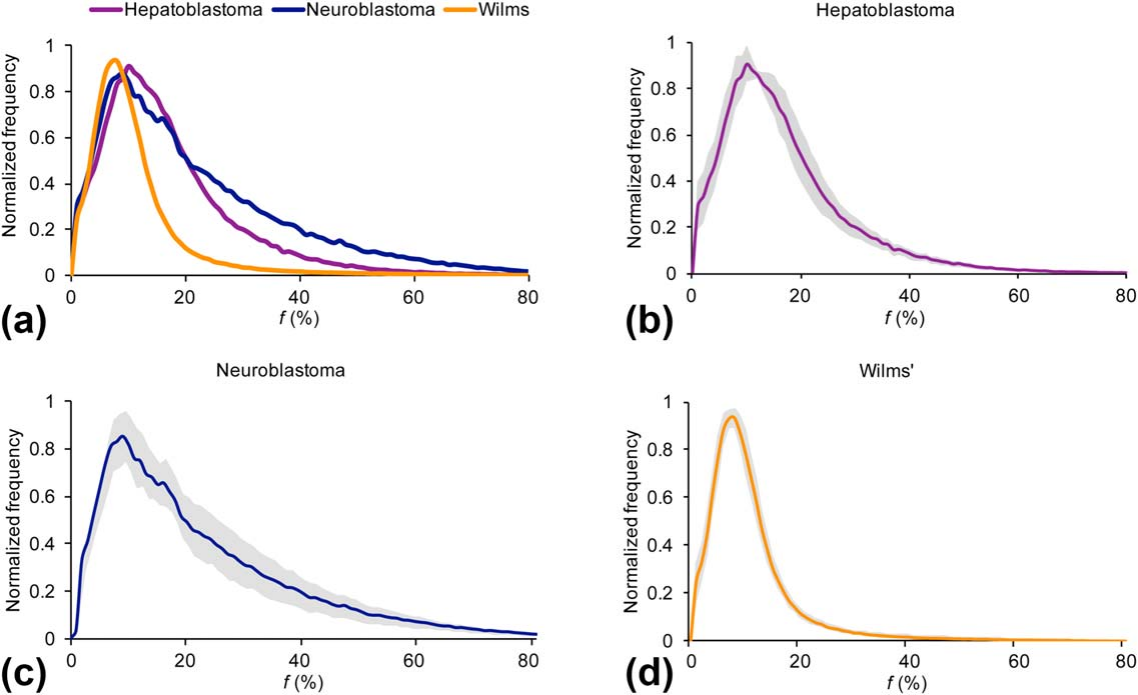
Average perfusion fraction, *f*, histograms. Histograms shown for **(a)** hepatoblastoma (*n* = 4), neuroblastoma (*n* = 11), and Wilms' tumor (*n* = 8). The **(b–d)** histograms indicate the SD (shown in gray) between cases in each major tumor group.

Normalized *f* histograms averaged for the malignant tumor types are shown in Fig. 3. The Wilms' and neuroblastoma histogram shapes demonstrated the differences seen in kurtosis and entropy between the tumor types. Kurtosis, which indicates the sharpness of a frequency‐distributed curve, was found to be higher and closer to normally distributed data in neuroblastoma in comparison to Wilms'. The higher entropy of neuroblastoma was observed as a more irregularly distributed histogram, with greater deviation seen between the cohort cases.

ROC analysis was performed for D* and *f* histogram parameters to study the diagnostic performance of discriminating neuroblastoma from Wilms' tumor, with the results summarized in Table 4. The D* histogram parameters mean, median, 75^th^/90^th^ percentiles, and skewness could discriminate the two tumor types with AUC values >0.900 (range, 0.909–0.935; *P* = 0.002–0.004). The *f* histogram parameters mean, median, 5^th^/25^th^/75^th^/90^th^, kurtosis, and entropy could discriminate Wilms' from neuroblastoma, with mean, median, 25^th^/75^th^/90^th^, and entropy demonstrating AUC values >0.900 (range, 0.938–1.00; *P* < 0.001–0.029).

**Table 4:** Diagnostic Performance of D* and *f* Histogram Parameters in Discriminating Wilms' Tumor From Neuroblastoma

Parameters		Mean	Median	5^th^ %	25^th^ %	75^th^ %	90^th^ %	Kurtosis	Skewness	Entropy
D*	AUC	0.909	0.909	0.506	0.675	0.909	0.935	0.571	0.909	0.532
	Confidence interval	0.739, 1.00	0.739, 1.00	0.177, 0.836	0.408, 0.942	0.739, 1.00	0.807, 1.00	0.285, 0.858	0.739, 1.00	0.254, 0.811
	Sensitivity %	90.9	90.9	81.8	81.8	90.9	90.9	63.6	90.9	45.5
	Specificity %	100	100	57.1	71.4	100	100	85.7	100	100
	*P* value	0.004	0.004	0.964	0.221	0.004	0.002	0.618	0.004	0.821
	Cutoff	17826	15636	5471	9612	22044	32376	2.22	7.60 × 10^−3^	9.62
*f*	AUC	1.00	1.00	0.784	0.938	1.00	0.966	0.886	0.523	0.977
	Confidence interval	1.00, 1.00	1.00, 1.00	0.574, 0.994	0.835, 1.00	1.00, 1.00	0.889, 1.00	0.676, 1.00	0.245, 0.801	0.920, 1.00
	Sensitivity %	100	100	50.0	75.0	100	87.5	87.5	50.0	87.5
	Specificity %	100	100	90.9	90.9	100	100	100	72.7	100
	*P* value	<0.001	<0.001	0.039	0.001	<0.001	0.001	0.005	0.869	0.029
	Cutoff	14.7	11.0	2.50	7.50	17.5	24.50	2.10	2.01 × 10^−1^	3.35

AUC is the area under the ROC curve. The units for mean, median, and percentiles of D* cutoff values are in ×10^−6^ mm^2^/s and *f* as a percentage (%).

**Figure 4:**
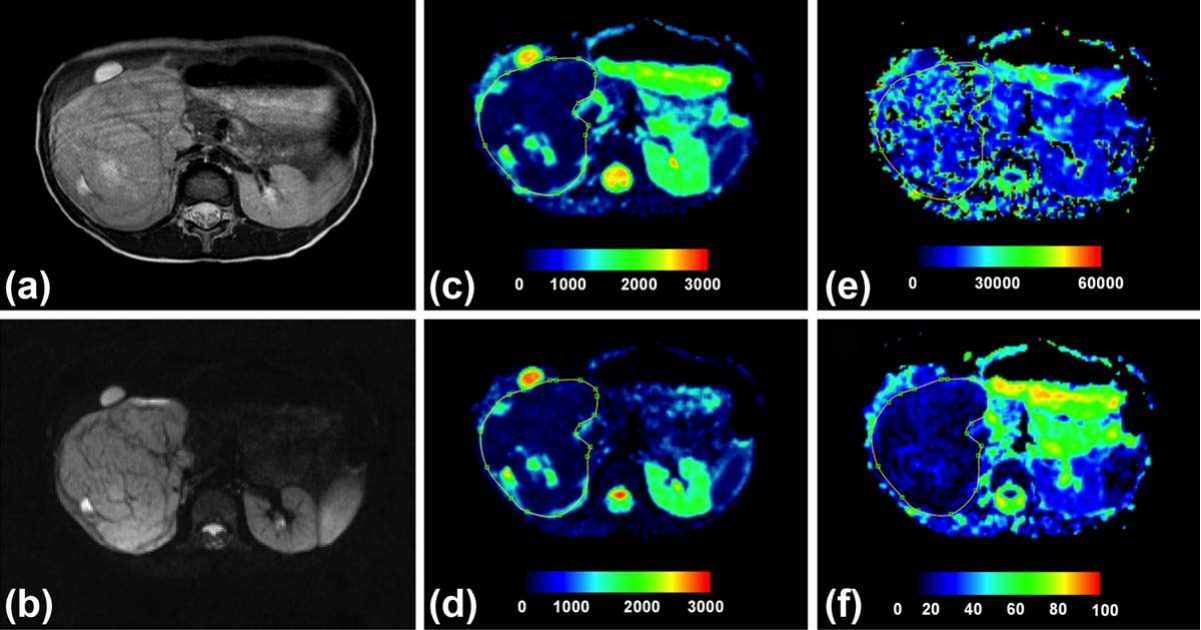
Histologically verified low‐risk Wilms' tumor. **(a)** T_2_‐weighted and **(b)** *b* = 150 axial images, and **(c–f)** parametric maps (ADC, D, D*, and *f*, respectively). Whole tumor ROI is shown drawn on the parametric maps. The calculated median values of ADC, D, D*, and *f* for the drawn ROI were 768 × 10^−6^ mm^2^/s, 677 × 10^−6^ mm^2^/s 13,726 × 10^−6^ mm^2^/s, and 10%, respectively.

**Figure 5:**
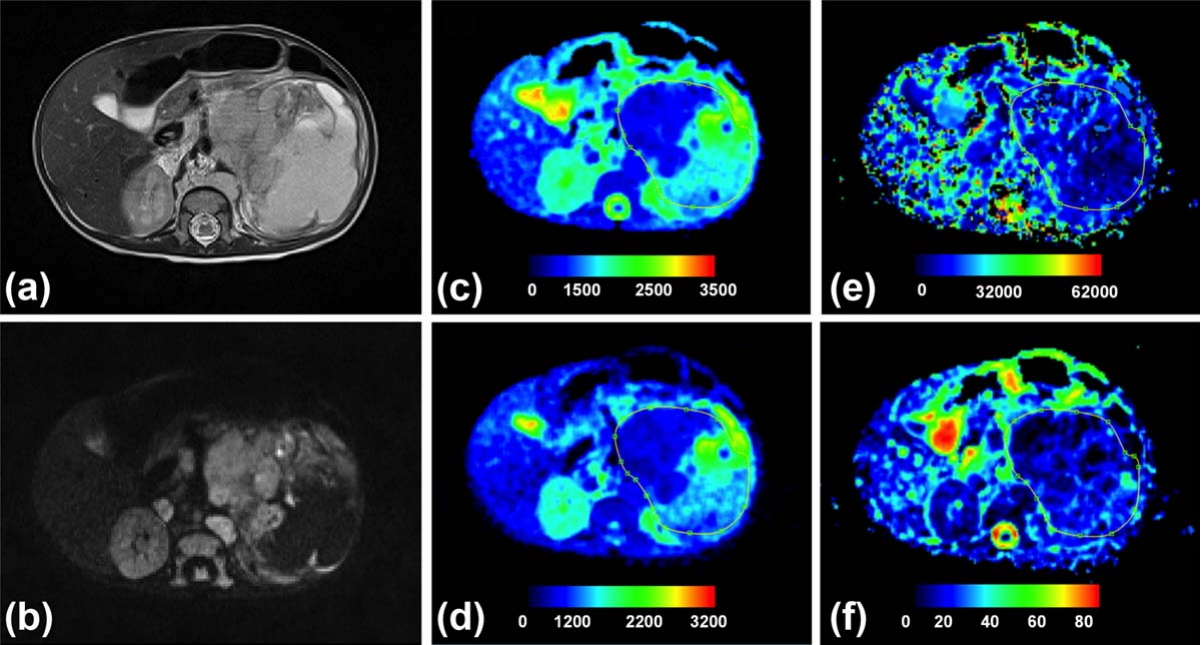
Histologically verified neuroblastoma (grade IV). **(a)** T_2_‐weighted and **(b)** *b* = 150 images, and **(c–f)** parametric maps (ADC, D, D*, and *f*, respectively). Whole tumor ROI is shown drawn on the parametric maps. The calculated median values of ADC, D, D*, and *f* for the drawn ROI were 1155 × 10^−6^ mm^2^/s, 703 × 10^−6^ mm^2^/s, 17,762 × 10^−6^ mm^2^/s, and 23%, respectively.

Representative parametric maps of Wilms' tumor and neuroblastoma are shown in Figs. 4 and 5, respectively. The ADC and *D* maps were similar in appearance, although *D* was generally lower in comparison to ADC and some regions suggested drops in DW signal when *b*‐value >0, seen as a lower intensity on *D* map in comparison to ADC. For both Wilms' and neuroblastoma, the D* maps appeared the most heterogeneous, indicating greater variability of values, which accounted for the high histogram entropy. In reference to the healthy kidneys seen on the *f* maps, a lower vascular character was suggested for the Wilms' case (Fig. 4), while more variability was observed for neuroblastoma (Fig. 5).

No correlation between IVIM and ADC parameters was found in the image regions where *f* was high (25–40%), suggesting that the higher *f* and D* values resulted from pathological origins rather than image artifacts. However, the overall voxelwise correlations in neuroblastoma and Wilms' cases demonstrated a negative correlation for *D* and *f*, while ADC and *f* showed positive correlation.

### Discrimination Between Benign and Malignant Lesions

The discrimination between benign and malignant lesions was possible with ADC, *D*, and D* parameters (Supplementary Table S1). The ADC, D, D*, and *f* for benign lesions were as follows: ADC = 1597 ± 484 (×10^−6^ mm^2^/s), *D* = 1552 ± 622 (×10^−6^ mm^2^/s), D* = 11,610 ± 3385 (×10^−6^ mm^2^/s), and *f* = 16 ± 6.7%. Both ADC and *D* were found to be significantly lower and D* significantly higher in malignant tumors. Most ADC histogram parameters demonstrated a significant difference with lower mean (*P* = 0.007), median (*P* = 0.001), 5^th^ percentile (*P* = 0.005), 25^th^ percentile (*P* = 0.007), 75^th^ percentile (*P* = 0.007) and higher kurtosis (*P* < 0.001), skewness (*P* < 0.001), and entropy (*P* = 0.036) for malignant tumors. The *D* parameter was only found to discriminate malignant tumors with lower median (*P* = 0.049), 25^th^ percentile (*P* = 0.045), and higher skewness (*P* = 0.018). While the *f* parameter was not found to not show difference between the benign and malignant lesions, the D* parameter demonstrated higher median (*P* = 0.039) and entropy (*P* = 0.002) in malignant tumors.

**Figure 6:**
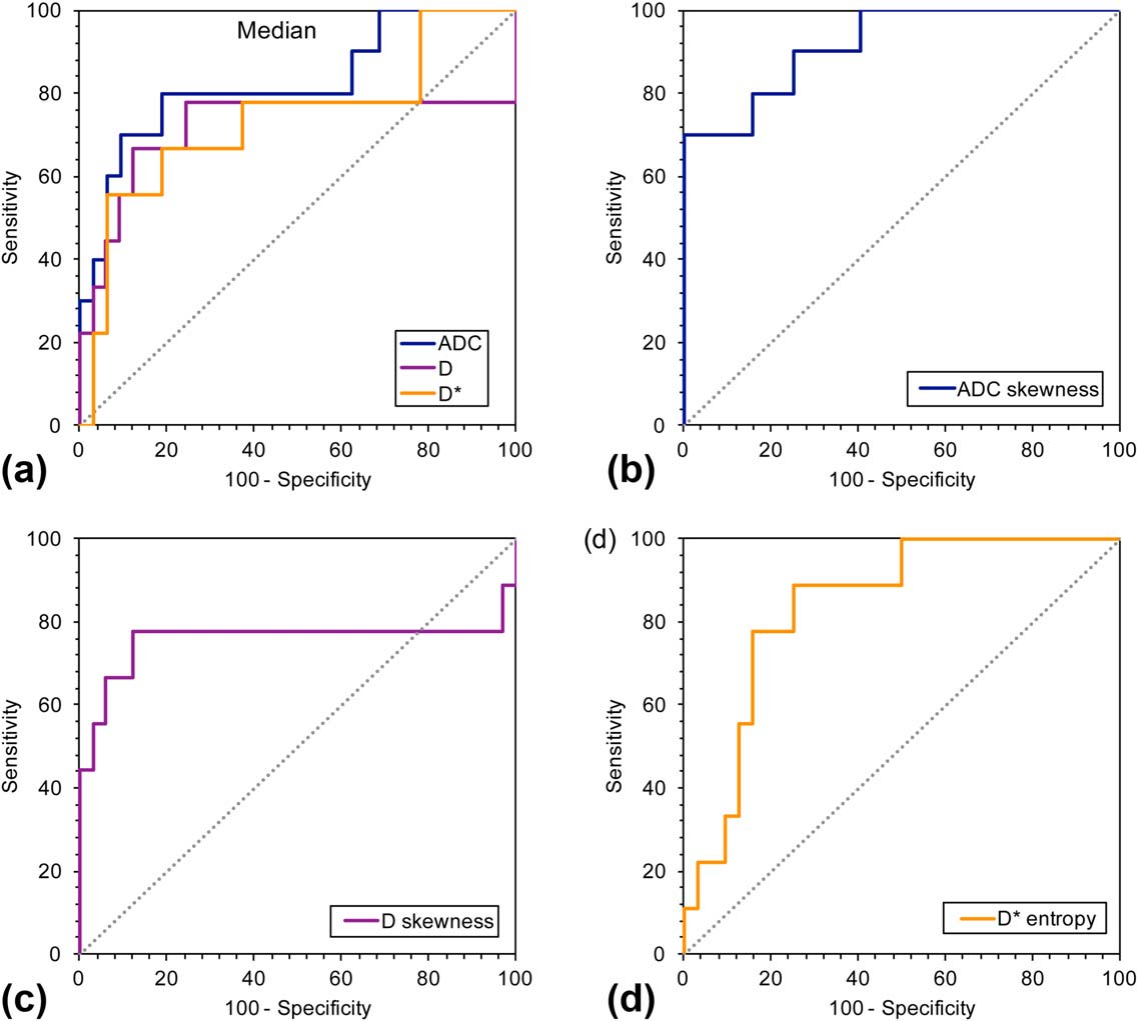
ROC curves for diffusion parameters to compare the performance in discriminating benign from malignant tumors. ROC curves for median **(a)** ADC, *D*, and D*, and the ROC curves with the highest AUC values for each parameter: **(b)** ADC skewness, **(c)** *D* skewness, and **(d)** D* entropy.

ROC analysis was performed for the diffusion parameters that demonstrated a significant difference between benign and malignant lesions (summarized results can be found in Supplementary Table S2). The ROC curves corresponding to median histogram values together with the highest resulting AUC values for ADC, *D*, and D* are presented in Fig. 6. The analysis showed that the median values of ADC 1219 × 10^−6^ mm^2^/s, *D* 1242 × 10^−6^ mm^2^/s, and D* 11,104 × 10^−6^ mm^2^/s were the most accurate cutoff levels, with sensitivity and specificity of 80.0% and 81.3% for ADC, 66.7% and 87.9% for *D*, and 55.6% and 93.8% for D*. The ADC was found to have better diagnostic performance than *D* or D* for discriminating benign from malignant tumors, with AUC values 0.825 (range, 0.659–0.991, *P* = 0.002) for ADC, 0.717 (range, 0.458–0.977, *P* = 0.048) for *D* and 0.736 (range, 0.528–0.945, *P* = 0.032) for D*.

The best diagnostic performance was achieved with skewness for ADC and *D* and entropy for D* with AUC values 0.919 (range, 0.823–1.00, *P* < 0.001), 0.758 (range, 0.495–1.00, *P* = 0.019), and 0.840 (range, 0.711–0.969, *P* = 0.002), respectively. The optimal cutoff values were ADC skewness: 3.30 × 10^−2^ (sensitivity 70.0%, specificity 100%), *D* skewness: 3.56 × 10^−2^ (sensitivity 77.8%, specificity 87.9%), and D* entropy 9.32 (sensitivity 88.9%, specificity 75.0%).

## Discussion

The present study investigated the diffusion and perfusion characteristics of pediatric abdominal tumors based on ADC and IVIM models. We have demonstrated ADC and IVIM‐derived histogram parameters to be helpful in discriminating between malignant neuroblastoma and Wilms' tumors as well as differentiating benign from malignant lesions. Distinctive IVIM‐derived D* and *f* histogram parameters were revealed for Wilms' tumors and neuroblastomas. No significant differences were observed with *D* or ADC histogram parameters for the individual tumor types. Discrimination of benign from malignant lesions was possible with the diffusion coefficients ADC, *D*, and D*, but not with *f* histogram parameters. These results suggest that the complex, heterogeneous structures of abdominal tumors can be characterized using histogram analysis, to potentially facilitate their noninvasive diagnosis.

The use of an IVIM model or IVIM histogram analysis for pediatric abdominal tumors has not been reported previously. The histogram approach has been increasingly used for heterogeneous tissues, allowing interpretation of the complex nature and features seen in tumors.^17^ Previous studies in adults have utilized histogram analysis of ADC and cerebral blood volume (CBV), which has been applied to a variety of scenarios including differentiation of true tumor progression from pseudoprogression,^28^, ^29^ response to chemotherapy,^30^ and grading of tumors.^31^, ^32^, ^33^ Most previous pediatric studies have reported mean or median ADC values based on a single section or slice, which does not address the heterogeneity seen in many abdominal tumors.^7^, ^8^

Our results indicate that IVIM‐derived histogram parameters may facilitate discrimination between malignant neuroblastoma and Wilms' tumors. Comparison of Wilms' tumors and neuroblastoma indicated significant differences in the tumor perfusion characteristics. The perfusion influenced parameters D* and *f* were found to be significant predictors of tumor type, while both ADC and *D* parameters were unable to discriminate between them. Although these tumors generally display different characteristic features, both in terms of clinical presentation^34^ and on conventional imaging,^35^, ^36^ differentiating Wilms' from neuroblastoma may cause diagnostic confusion in the case of intrarenal neuroblastoma,^37^, ^38^ or if the tumor involves the adrenal gland.^39^ Neuroblastomas commonly encase vascular structures,^34^ which could be observed as an increase in D* and *f* parameters. The lack of correlation between IVIM and ADC parameters in regions where *f* was 20–40% suggests that higher *f* and D* values resulted from pathology rather than image artifact. This finding is of clinical importance, as it could potentially be applied to other tumors, which are more difficult to discriminate clinically, such as distinguishing Wilms' from renal rhabdoid or clear‐cell sarcoma of the kidney, or mesoblastic nephroma. This is particularly relevant given the recommendation to avoid pretreatment biopsy in these tumors.^40^

In addition to the higher values of the IVIM perfusion parameters, higher skewness for D* and higher entropy for *f* were observed in neuroblastoma. This is suggestive of a more irregular and heterogeneous vasculature of neuroblastoma compared to Wilms' tumors. Diagnostic performance with sensitivity and specificity above 85% was achieved with D* mean, median, 75^th^/90^th^ percentiles, and skewness, and with *f* using mean, median, 75^th^/90^th^ percentiles, kurtosis, and entropy.

Cellularity and vascularity have been hypothesized to increase in a similar manner, corresponding to a decrease in ADC and increase in *f*. Interestingly, voxelwise correlations in neuroblastoma and Wilms' cases demonstrated this to be true for *D* and *f*, with negative correlation shown, whereas ADC and *f* showed positive correlation. This agrees with the IVIM model and the proposed “true” tissue coefficient *D*, and the contribution of both diffusion and vascular characteristics to the measure of ADC.

A review of our results in a clinical context revealed the interesting observations that tumors following an unusual clinical course often had *f* values differing from others in the same tumor group. Tumors behaving particularly aggressively tended to have higher *f* values, whereas those following a more indolent course had lower *f* values. The mean *f* value for Wilms' tumors in our cohort was 11.0 ± 0.8%; the patient with the highest mean *f* value (14.4%) died following a very aggressive disease process with multiple relapses. Rhabdoid tumors are typically aggressive malignancies with poor survival. The two patients in our cohort with rhabdoid tumors had mean *f* values of 23.2% and 8.4%, the former died 3 months after presentation, whereas the latter remains in remission 2 years following completion of treatment. This is suggestive of *f* being a potential prognostic biomarker, with high‐risk tumors having high *f* values. This is biologically plausible, with increased tumor vascularity reflected through an increase in *f*. Although our numbers are too small to draw firm conclusions, this interesting observation deserves further exploration in a larger cohort.

Many of the benign lesion types included in this study can be commonly diagnosed on conventional MRI. However, the benign cases in our cohort were a subset of these that were suspected for malignancy and their diagnosis based on clinical information and conventional MRI was not possible. Therefore, all cases required histological verification. Additional imaging methods such as DW‐MRI can provide particularly useful information for diagnostically difficult cases. In our cohort, the discrimination of benign from malignant lesions was feasible with the histogram parameters of ADC, *D*, and D*. Of these parameters, the best performance was observed with the ADC, which performed well across the percentiles (≤75^th^ percentile). It is possible that ADC, influenced by both *D* and D*, has a higher diagnostic performance due to the combinations of diffusion and perfusion characteristics into a single parameter. Interestingly, ADC and *D* were lower in malignant tumors, while D* was higher. Quantitative assessment of ADC is simpler than performing IVIM, and would be feasible to incorporate into clinical practice, with considerable potential to improve patient care. The robust performance of ADC across the percentiles also suggests that the use of histogram analysis might be less relevant, and the use of mean or median might be sufficient for discrimination of benign from malignant lesions. This finding should be tested with a cohort including a larger number of benign cases.

The *f* parameter failed to reach significance in discrimination of benign from malignant tumors. A wide range of *f* values was observed for the benign cohort. This may reflect differences in vasculature rather than cellularity, particularly as benign lesions evaluated included a hemangioma and vascular malformation. Although the *f* parameter may not determine benign from malignant cases, it may be useful in evaluating the vascular environment within lesions.

Our study has several limitations. First, the sample size was relatively small for the comparison of individual tumor types. However, the preliminary results are promising and suggested discrimination may be possible, although further validation is required in a larger cohort. Second, no T_2_ correction^41^ was applied to the perfusion fraction, and therefore the values could have been affected by the T_2_ relaxation times of blood and tissue. Third, the group sizes were unbalanced for discrimination of benign from malignant tumor types and further larger and multicenter studies are required to confirm these results. Finally, while the IVIM model is becoming more popular in abdominal applications, the biophysical origins of the IVIM parameters require further exploration and justification. Promising results were shown in a previous study of the pancreas, which was able to verify the vascular contribution to the diffusion signal by varying the echo time of the MR acquisition.^41^

In conclusion, our results suggest that IVIM parameters and their histogram analysis can provide useful insight into the complex structures of pediatric abdominal tumors. The use of multi *b*‐value DW‐MRI allowed the computation of IVIM parameters, and the whole‐tumor ROI approach ensured that the heterogeneity of the tumors was taken into consideration. Our initial results in the childhood abdominal tumors suggest that the use of IVIM perfusion parameters, and in particular the perfusion fraction, could facilitate the diagnosis of individual tumor types, and therefore provide a set of noninvasive imaging biomarkers for their characterization.

## Supporting information

Additional supporting information may be found in the online version of this article

Supporting Information
