# Supplementary material for: Diffusion‐weighted MRI and intravoxel incoherent motion model for diagnosis of pediatric solid abdominal tumors
Source: J Magn Reson Imaging. 2017 Nov 21;47(6):1475–86. doi: 10.1002/jmri.25901 (PMC6001424; doi:10.1002/jmri.25901)
Supplement: Supplementary file 1 — Supporting Information [file JMRI-47-1475-s001.pdf]

Supplementary Table S1. Comparison of benign ($n$ = 10) and malignant ($n$ = 32) lesions with ADC, $D$, $D^*$ and $f$ histogram parameters.

| Parameter | Mann-Whitney test | | | |
|---|---|---|---|---|
| | ADC | $D$ | $D^*$ | $f$ |
| Mean | 0.007* | 0.070 | 0.127 | 0.390 |
| Median | 0.001* | 0.049* | 0.039* | 0.320 |
| $5^{th}$ percentile | 0.005* | 0.056 | 0.135 | 0.919 |
| $25^{th}$ percentile | 0.002* | 0.045* | 0.092 | 0.248 |
| $75^{th}$ percentile | 0.007* | 0.052 | 0.127 | 0.494 |
| $90^{th}$ percentile | 0.202 | 0.112 | 0.259 | 0.760 |
| Kurtosis | < 0.001* | 0.740 | 0.247 | 0.631 |
| Skewness | < 0.001* | 0.018* | 0.647 | 0.122 |
| Entropy | 0.036* | 0.081 | 0.002* | 0.965 |

Note: * indicates $P$ value of a significant difference.

Supplementary Table S2. Comparison of benign (*n* = 10) and malignant (*n* = 32) lesions with ADC, *D*, *D** and *f* histogram parameters with ROC analysis results.

| Parameters | | Mean | Median | $5^{th}$ % | $25^{th}$ % | $75^{th}$ % | $90^{th}$ % | Kurtosis | Skewness | Entropy |
|---|---|---|---|---|---|---|---|---|---|---|
| ADC | AUC | 0.781 | 0.825 | 0.788 | 0.819 | 0.778 | 0.638 | 0.875 | 0.919 | 0.722 |
| | Confidence interval | 0.601, 0.962 | 0.659, 0.991 | 0.488, 0.987 | 0.646, 0.992 | 0.610, 0.946 | 0.439, 0.836 | 0.738, 1.000 | 0.823, 1.000 | 0.513, 0.931 |
| | Sensitivity % | 70.0 | 80.0 | 70.0 | 70.0 | 70.0 | 90.0 | 80.0 | 70.0 | 60.0 |
| | Specificity % | 87.5 | 81.3 | 90.6 | 93.7 | 81.2 | 43.7 | 93.7 | 100 | 87.5 |
| | *P* value | 0.008* | 0.002* | 0.007* | 0.003* | 0.009* | 0.194 | <0.001* | <0.001* | 0.036* |
| | Cutoff | 1439 | 1219 | 951 | 1310 | 1565 | 1605 | 2.10 | $3.30 \times 10^{-2}$ | 6.55 |
| *D* | AUC | 0.700 | 0.717 | 0.710 | 0.721 | 0.714 | 0.677 | 0.539 | 0.758 | 0.694 |
| | Confidence interval | 0.451, 0.950 | 0.458, 0.977 | 0.451, 0.970 | 0.459, 0.983 | 0.487, 0.941 | 0.471, 0.883 | 0.296, 0.782 | 0.495, 1.000 | 0.493, 0.894 |
| | Sensitivity % | 66.7 | 66.7 | 77.8 | 66.7 | 66.7 | 88.9 | 33.3 | 77.8 | 77.8 |
| | Specificity % | 84.8 | 87.9 | 27.7 | 90.9 | 81.8 | 51.5 | 90.9 | 87.9 | 66.7 |
| | *P* value | 0.068 | 0.048* | 0.055 | 0.045* | 0.052 | 0.108 | 0.724 | 0.019* | 0.078 |
| | Cutoff | 1232 | 1242 | 587 | 1117 | 1375 | 1421 | 1.93 | $3.56 \times 10^{-2}$ | 6.98 |
| *D** | AUC | 0.681 | 0.736 | 0.681 | 0.701 | 0.681 | 0.639 | 0.628 | 0.569 | 0.840 |
| | Confidence interval | 0.442, 0.919 | 0.528, 0.945 | 0.481, 0.880 | 0.495, 0.907 | 0.435, 0.926 | 0.387, 0.891 | 0.383, 0.874 | 0.342, 0.797 | 0.711, 0.969 |
| | Sensitivity % | 55.6 | 55.6 | 88.9 | 66.7 | 55.6 | 44.4 | 55.6 | 77.8 | 88.9 |
| | Specificity % | 87.5 | 93.8 | 469 | 81.2 | 87.5 | 90.6 | 90.6 | 46.9 | 75.0 |
| | *P* value | 0.101 | 0.032* | 0.101 | 0.068 | 0.101 | 0.208 | 0.244 | 0.529 | 0.002* |
| | Cutoff | 13700 | 11104 | 5206 | 8473 | 16642 | 23690 | 2.19 | $7.61 \times 10^{-3}$ | 9.32 |
| *f* | AUC | 0.594 | 0.605 | 0.513 | 0.625 | 0.573 | 0.534 | 0.553 | 0.666 | 0.506 |
| | Confidence interval | 0.372, 0.815 | 0.368, 0.841 | 0.291, 0.734 | 0.392, 0.858 | 0.354, 0.793 | 0.325, 0.744 | 0.369, 0.737 | 0.470, 0.861 | 0.303, 0.710 |
| | Sensitivity % | 40.0 | 40.0 | 30.0 | 40.0 | 70.0 | 60.0 | 80.0 | 90.0 | 70.0 |
| | Specificity % | 87.5 | 93.7 | 93.7 | 87.5 | 59.4 | 62.5 | 50.0 | 46.9 | 53.1 |
| | *P* value | 0.376 | 0.323 | 0.906 | 0.238 | 0.488 | 0.745 | 0.616 | 0.118 | 0.953 |
| | Cutoff | 22.7 | 19.5 | 5.50 | 10.5 | 21.5 | 36.0 | 2.18 | $2.06 \times 10^{-1}$ | 3.52 |

Note: * indicates *P* value of a significant difference. AUC is the area under the ROC curve. The units for mean, median and percentiles of ADC, *D* and *D** cutoff values are in $\times 10^{-6}$ $mm^2/s$ and *f* as a percentage (%).
